# Cognitive Status Predicts Return to Functional Independence After Minor Stroke: A Decision Tree Analysis

**DOI:** 10.3389/fneur.2022.833020

**Published:** 2022-02-17

**Authors:** Mirjam R. Heldner, Caroline Chalfine, Marion Houot, Roza M. Umarova, Jan Rosner, Julian Lippert, Laura Gallucci, Anne Leger, Flore Baronnet, Yves Samson, Charlotte Rosso

**Affiliations:** ^1^Department of Neurology, Inselspital, University Hospital and University of Bern, Bern, Switzerland; ^2^Assistance Publique – Hôpitaux de Paris (APHP) Service de Soins de Suite et Réadaptation, Hôpital Pitié-Salpêtrière, Paris, France; ^3^Assistance Publique – Hôpitaux de Paris (APHP) Centre d'Investigations Cliniques de Neurosciences, Hôpital Pitié-Salpêtrière, Paris, France; ^4^Inserm U 1127, CNRS UMR 7225, Sorbonne Université, UPMC Univ Paris 06 UMR S 1127, Institut du Cerveau et de la Moelle épinière (ICM), Paris, France; ^5^Spinal Cord Injury Center, Balgrist University Hospital, University of Zurich, Zurich, Switzerland; ^6^STARE Team, iCRIN, Institut du Cerveau et de la Moelle épinière (ICM), Paris, France; ^7^APHP-Urgences Cérébro-Vasculaires, Hôpital Pitié-Salpêtrière, Paris, France

**Keywords:** minor stroke, cognition, prediction, CART, prognosis

## Abstract

About two-thirds of patients with minor strokes are discharged home. However, these patients may have difficulties returning to their usual living activities. To investigate the factors associated with successful home discharge, our aim was to provide a decision tree (based on clinical data) that could identify if a patient discharged home could return to pre-stroke activities and to perform an external validation of this decision tree on an independent cohort. Two cohorts of patients with minor strokes gathered from stroke registries at the Hôpital Pitié-Salpêtrière and University Hospital Bern were included in this study (*n* = 105 for the construction cohort coming from France; *n* = 100 for the second cohort coming from Switzerland). The decision tree was built using the classification and regression tree (CART) analysis on the construction cohort. It was then applied to the validation cohort. Accuracy, sensitivity, specificity, false positive, and false-negative rates were reported for both cohorts. In the construction cohort, 60 patients (57%) returned to their usual, pre-stroke level of independence. The CART analysis produced a decision tree with the Montreal Cognitive Assessment (MoCA) as the first decision point, followed by discharge NIHSS score or age, and then by the occupational status. The overall prediction accuracy to the favorable outcome was 80% in the construction cohort and reached 72% accuracy in the validation cohort. This decision tree highlighted the role of cognitive function as a crucial factor for patients to return to their usual activities after a minor stroke. The algorithm may help clinicians to tailor planning of patients' discharge.

## Introduction

About two-third of the patints with minor strokee (NIHSS ≤ 5) are discharged home. However, these patients may have difficulties in their usual activities, such as instrumental or even in basic activities of daily living (ADLs) (1, 2).

Although various studies have assessed predictors of home discharge of patients with stroke (3–5), few have assessed whether these patients actually returned to their pre-stroke activities. This is, however, a crucial factor as it influences the patients' need for support and a potential requirement of the ongoing outpatient rehabilitation. Therefore, knowing whether patients will return to their daily routines helps to optimally allocate resources and therapy strategies, ultimately improving patient outcomes.

One recent review (3) focused on predictive models related to the need for supportive services in patients discharged home with various diagnoses (stroke, general medicine ward). Several studies have shown that the severity of initial disability within the first days post-stroke, combined with age, was an important indicator of outcome at 3-months after stroke (6). However, it is unclear whether these findings could be extrapolated to patients with minor strokes. It has been shown that neurological deficits in the baseline NIHSS do not predict functional outcomes (7). Indeed, the NIHSS and other predictors, such as the presence of a proximal vessel occlusion assessed in prior studies, can underestimate the full scale of sequelae post-stroke. Cognitive deficits, particularly visuospatial or executive dysfunctions occurring after a minor stroke, might limit functional independence (8) and should be evaluated early during the acute phase with a simple score, such as the MoCA (9). The MoCA is a screening tool for cognitive dysfunction testing executive functions, visuospatial abilities, language, memory, and abstract reasoning. Administration of this screening tool was shown to be feasible and robust in an acute stroke setting.

In this study, we aimed to provide a predictive tool using sociodemographic data, neurological status as indexed by the NIHSS, and cognitive function derived from the MoCA as factors to identify if a patient discharged home could return to carry his or her prestroke usual activities. Second, we sought to externally validate this tool on an independent external cohort. We hypothesized that cognitive dysfunction measured with the MoCA at discharge will play a major role for 3-months excellent outcome besides age and NIHSS score.

As the issue of implementation of predictive models in clinical routine is tremendously challenging (10–12), and as models must be user-friendly to be applied through a simplified interface, we decided to use classification and regression tree (CART) analysis to generate a decision tree in this prediction.

## Methods

### Patients

For the construction cohort, consecutive patients were prospectively screened from January 2017 until February 2020 at the Stroke Emergency department (Hôpital de la Pitié Salpêtrière, Paris, France). Inclusion criteria were (1) any stroke event in patients admitted to the department, (2) discharge to home at the end of the hospitalization, (3) living in Paris or its surroundings, (4) an NIHSS ≤ 5 at admission. (5) Exclusion criteria were: (i) death during the 3-month follow-up, and (ii) missing data on the variables of interest.

All imaging and clinical data were obtained during routine clinical workup in the stroke center. According to French legislation, informed consent was a letter where the participant did not sign but his physician did (non-opposition letter). GDPR regulatory rules (General Data Protection Regulation) were followed. The local ethics committee approved the study.

For the validation cohort, 101 patients from the Bernese stroke Registry with the same inclusion/exclusion criteria were analyzed. In Bern, patients or their relatives gave informed consent for treatment and study participation. The study was approved by the local ethics committee of the canton of Bern (KEK 231/14).

Patients were selected from the time period preceding the COVID-19 pandemic since the outbreak disturbed the clinical trajectories of the patients in our units.

### Datasets

#### Pitié-Salpêtrière Cohort

Data collected during the hospitalization were demographic (age, gender), data related to stroke event: type (ischemic, hemorrhagic strokes or cerebral venous thrombosis), affected hemisphere (left, right), presence of motor or sensory symptoms, NIHSS score at admission to the stroke unit and at discharge, modified Rankin Scale (mRS) at discharge, and MoCA score before discharge (usually in the last two days before discharge).

Socio-professional characteristics were recorded, such as household situation (living with someone, living alone but supported by proxies, or living alone), occupational status (employed, retired, unemployed), need for technical support (yes or no) or caregivers (yes or no) in usual activities, and pre-stroke independence assessed by the mRS.

At 3 months, patients were examined on their follow-up visit and explicitly asked whether they returned to their pre-stroke normal life (usual activities, even if they still experienced symptoms) meaning whether they had an mRS of 0 or 1. Patients who returned to their activities of pre-stroke level were assigned to the favorable outcome group and patients who failed were assigned to the poor outcome group.

#### Bernese Cohort

The Bernese stroke registry is a prospectively collected database, which has been described previously (13). In brief, all patients who suffered an acute stroke and were admitted to the University Hospital Bern are included in this database. All variables, described above were also routinely collected in the Bernese Cohort.

### Statistics

The normality of data was tested with the Shapiro–Wilk test. Descriptive statistics consisted then of mean and standard deviations (SD). Comparisons of proportions were determined by a chi-squared test, and the quantitative variables were compared by *t*-tests. These analyses were performed using MedCalc software (version 12.5.0, Belgium, 2013). There were no missing data in both cohorts.

The CART analysis was carried out with R studio (version 1.1.463) in the construction cohort to determine which factors best predicted whether patients returned to their pre-stroke usual activities 3-months after a stroke (see Supplementary Material for details on CART).

Here, the target outcome corresponds to patients with an mRS of 0 or 1 (as a binary variable) 3-months after a stroke. The explanatory variables were age, gender, pre-stroke mRS, affected hemisphere, type of stroke, presence of motor or sensory symptoms, occupational status, household situation, need of assistance (technical, human or neither), NIHSS score on admission and at discharge, and MoCA and mRS at discharge. Before entering these variables into the CART analysis, we performed a principal component analysis (PCA) to identify if some of these variables covaried together and could be removed. The PCA correlation circle (Supplementary Figure 1) indicated that NIHSS at admission and discharge covaried together as well as pre-stroke mRS and need of assistance. Thus, admission NIHSS and need of assistance were removed from the CART analysis because of their redundancy and the decision tree was generated.

The decision tree was then applied to the validation cohort. Accuracy, sensitivity, specificity, false-positive, and false-negative rates were reported for both cohorts.

## Results

### Construction of the Decision Tree

**Figure 1:**
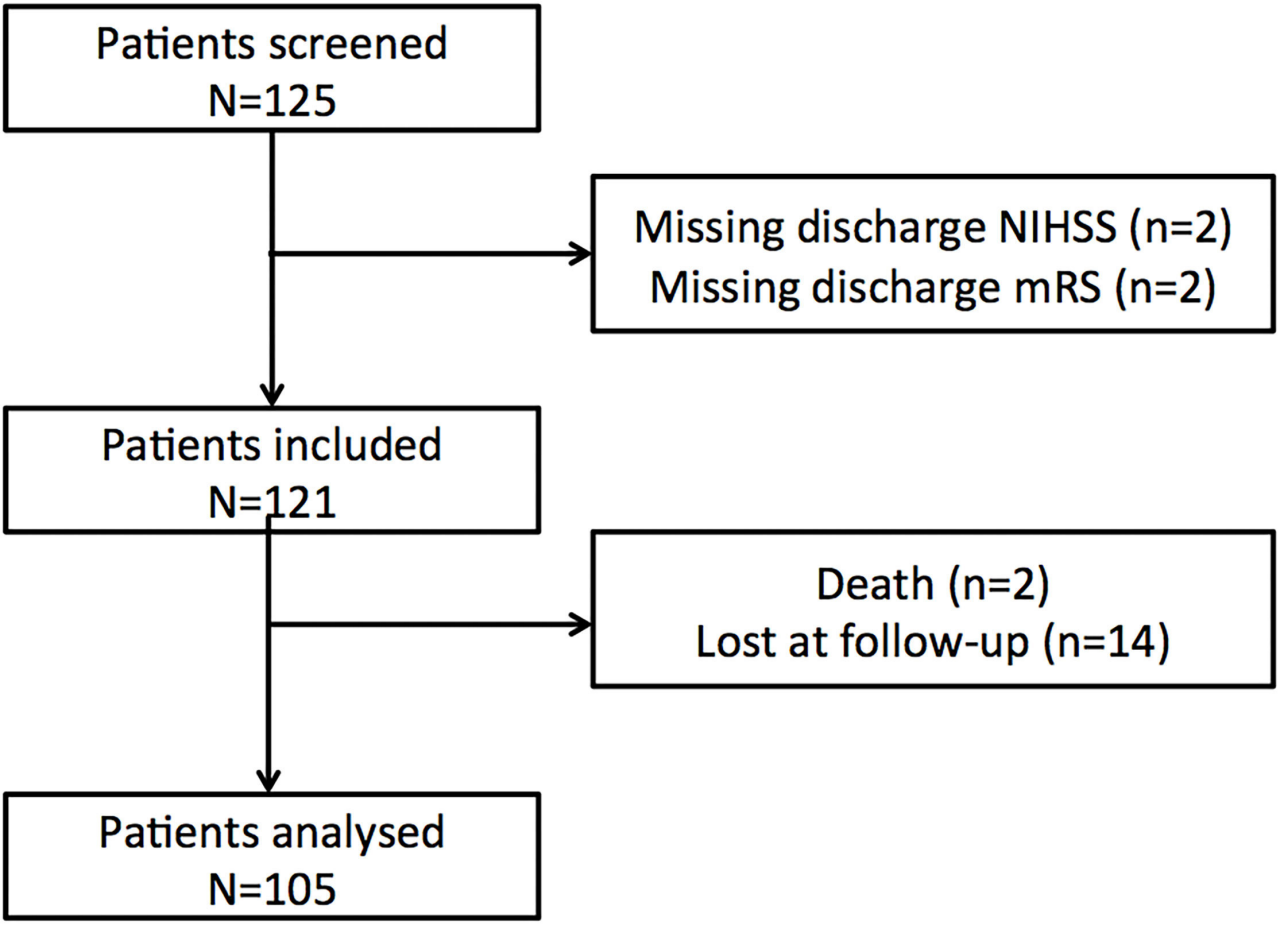
Flowchart of the training cohort.

One hundred and twenty-five patients were screened in the Pitié-Salpêtrière registry. Twenty patients were discarded (Figure 1, flow chart) and 105 patients were included in the final analysis. Baseline characteristics for the whole population and the favorable/poor outcome groups are summarized in Tables 1, 2. At 3 months, 60 patients (57%) returned to their pre-stroke routine life (mRS 0–1) but 45 (43%) failed to do so (mRS >1). Forty-three patients were oriented after home discharge either to a physiotherapist or a speech therapist. The good outcome rate was similar between patients with rehabilitation (22/43, 51.1%) versus those without (38/62, 61.3%, *p* = 0.40).

**Table 1:** Baseline characteristics of the construction cohort.

	**All patients** ***N*** **= 105**	**Favorable outcome** ***N*** **= 60**	**Poor outcome** ***N*** **= 45**	***p*** **-value**
Age (years), mean ± SD	65.5 ± 15.7	65.5 ± 17.0	65.4 ± 14.1	0.97
Gender, Men (*n*, %)	61 (58%)	37 (61%)	24 (40%)	0.53
Cardiovascular risk factors (*n*, %)				
Hypertension	63 (60%)	31 (52%)	32 (71%)	0.19
Hypercholesterolemia	60 (57%)	36 (60%)	24 (53%)	0.78
Diabetes	18 (17%)	11 (18%)	7 (16%)	0.59
Smoking	26 (25%)	20 (33%)	6 (13%)	0.66
Atrial Fibrillation	16 (15%)	8 (13%)	8 (17%)	0.63
Stroke Type (*n*, %) Ischemic, Hemorrhage, VT^a^	93/10/2 89%/9%/2%	53/5/2 88%/9%/3%	40/5/0 89%/11%/0%	0.88
Hemisphere affected (*n*, %) (left/right/both)	48/43/14 45%/41%/13%	26/30/4 43%/50%/7%	22/13/10 49%/29%/22%	0.68
Household situation (*n*, %) not living alone/living alone but proxies/alone	93/10/2 89%/9%/2%	40/15/5 67%/25%/8%	27/13/5 60%/29%/10%	0.59
Occupational status (*n*, %) Employed/Retired/unemployed	43/55/7 41%52%/7%	23/34/3 38%/55%/5%	20/21/4 44%/47%/9%	0.67
Discharge treatment (*n*, %)				
Antiplatelet agents	69 (66%)	40 (67%)	29 (64%)	0.91
Anticoagulants	19 (18%)	10 (17%)	9 (20%)	0.89
Length of stay (days) mean ± SD	7.1 ± 3.8	7.1 ± 3.6	7.0 ± 4.1	0.87

a *VT, Venous thrombosis; the p-value refers to the comparison between the favorable vs. poor outcome group*.

**Table 2:** Clinical symptoms and scores of the patients of the construction cohort.

	**All patients** ***N*** **= 105**	**Favorable outcome** ***N*** **= 60**	**Poor outcome** ***N*** **= 45**	***p*** **-value**
Independency pre-stroke (*n*, % of mRS^*a*^ 0-1)	97 (93%)	56 (93%)	41 (91%)	0.99
Admission NIHSS^b^ score, mean ± SD	1.3 ± 1.3	1.0 ± 1.2	1.6 ± 1.4	0.02
MoCA score, mean ± SD	23 ± 5	24.5 ± 4.3	21.5 ± 4.4	<0.001
Motor symptoms (yes, *N*, %)	21 (20%)	11 (18%)	10 (22%)	0.79
Sensory symptoms (yes, *N*, %)	22 (21%)	9 (15%)	13 (29%)	0.13
Discharge NIHSS^b^ score				
- mean/SD	0.8 ± 1.5	0.6 ± 1.1	0.9 ± 0.9	0.28
- N, % patients with a score of 0	55 (52%)	37 (61%)	18 (40%)	0.05
Discharge mRS^a^ 0-1 (*N*, %)	60 (57%)	40 (67%)	20 (44%)	0.03

a *mRS, modified Rankin Scale*;

b *NIHSS, National Institute of Health Stroke Scale*.

**Figure 2:**
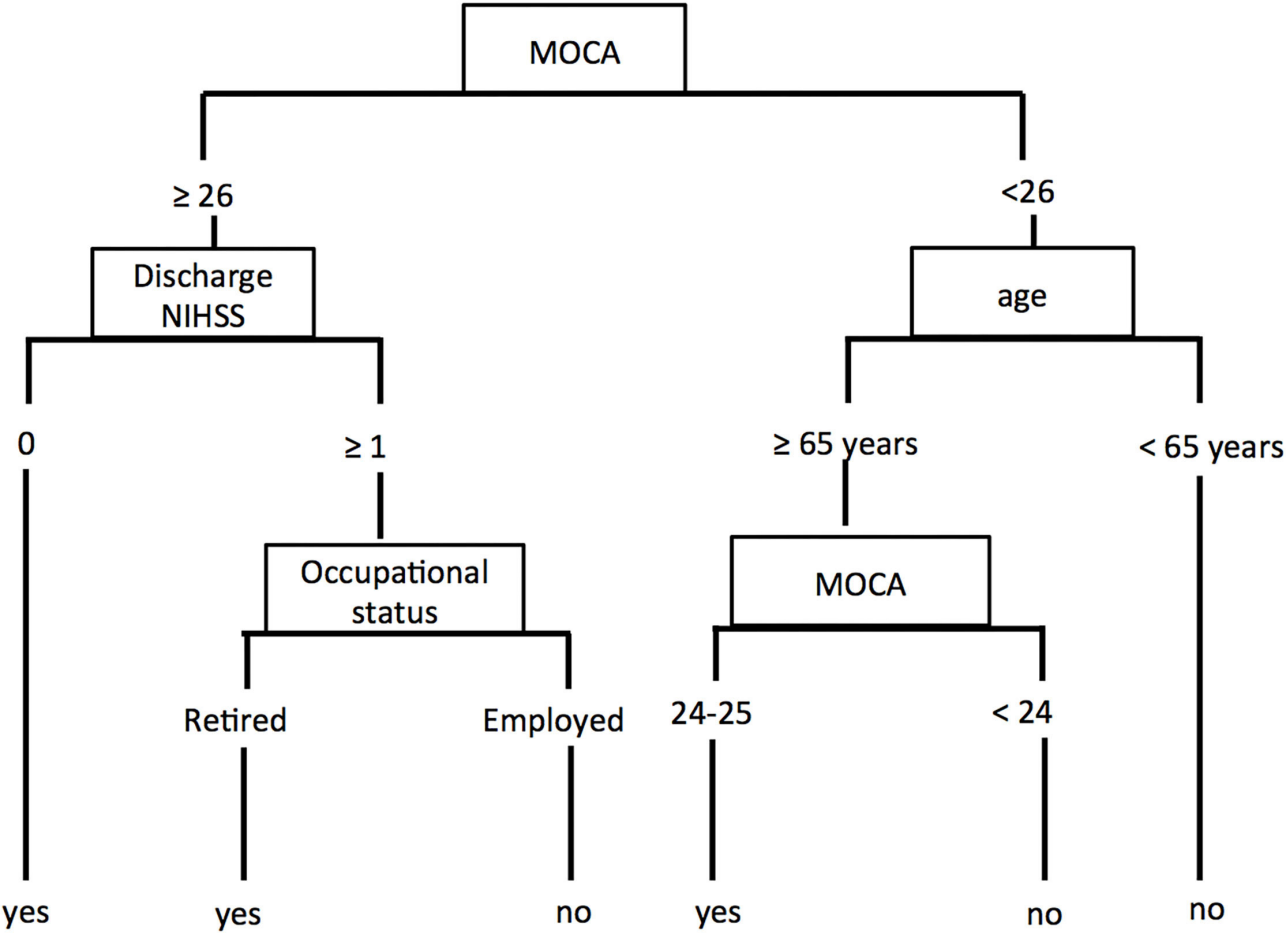
Decision tree to predict favorable outcome.

The CART analysis produced a decision tree (Figure 2) with the MoCA as the first decision point, followed by discharge NIHSS score or age and then by the occupational status.

Patients with a MoCA ≥ 26 and NIHSS score of 0 at discharge were predicted to have a favorable outcome. Patients with a MoCA ≥ 26 and NIHSS score > 0 at discharge reached a favorable outcome if they were retired, otherwise, they did not return to their full pre-stroke usual activities.

Patients with a MoCA <26 and younger than 65 years-old were predicted to have a poor outcome. If they were 65 years-old or older, their outcome depended on the score of the MoCA with a cutoff of 24 for returning or not to their usual pre-stroke activities.

The overall prediction accuracy for a favorable outcome was 80% (84/105), sensitivity was 71.6% (43/60), and specificity was 91% (41/45). Errors of outcome prediction represented 20% of the patients. Four (3.8%) were false positive (predicted as a favorable outcome but did not reach it) and 17 (16.2%) were false negative (predicted as a poor outcome but recovered better than expected).

### Validation of the Decision Tree

One hundred and one patients from the Bernese stroke Registry were recruited in the study in the time period between July 2019 and February 2020. One hundred were included after the exclusion of one patient who died during the 3-month follow-up time period. Characteristics of patients are provided in Table 3. Patients in this validation cohort were older than those of the construction cohort (*p* < 0.001), showed more severe stroke symptoms on the admission NIHSS (*p* < 0.001), and were less independent (mRS 0-1) before the stroke event (*p* < 0.001). At 3 months, 39 patients (39%) returned to their usual pre-stroke activities.

**Table 3:** Characteristics of the validation cohort from the Bernese registry (*n* = 49).

	**Patients**
Age (years) (mean ± SD)	72.4 ± 13.8
Gender, Men (*n*, %)	63% (63)
Cardiovascular risk factors (*n*,%)	
Hypertension	65% (65)
Hypercholesterolemia	54% (54)
Diabetes	35% (35)
Tobacco	21% (21)
Household situation (*n*, %)
not living alone/living alone but proxies/alone	89%/9%/2% 89/9/2
Occupational status (*n*, %)
Employed/retired/unemployed	22%/76% /2% 22/76/2
mRS† pre-stroke (*n*, % of mRS 0–1)	52% (52)
Admission NIHSS score (mean ± SD)	2.0 ± 1.7
MoCA score (mean ± SD)	23.8 ± 4.2
Discharge NIHSS score	
- mean/SD	1.5 ± 1.9
- *N*, % patients with a score of 0	39% (39)

The decision tree applied to this population reached a 72% (72/100) accuracy, 76.9% sensitivity (30/39), and 68.9% specificity (42/61). False negative and positive rates were 9% (*n* = 9) and 19% (*n* = 19), respectively.

As the number of patients with a pre-stroke mRS >1 was higher in the Bernese vs. the French cohort, we did a subgroup analysis in both cohorts for patients with a pre-stroke mRS 0–1 (see Supplementary Analysis).

## Discussion

In our study, approximately half of the patients with minor stroke who were discharged home did not return to their usual pre-stroke activities. We proposed a decision tree which was efficient, accurate, and simple for identifying patients who are at risk to remain dependent after home discharge 3 months after a stroke. Moreover, we demonstrated with an external validation that this decision tree was robust and could be applied in other centers.

### Outcome of Minor Stroke

As clinicians, we consider most minor stroke survivors to have non-disabling functional deficits because of their low NIHSS score, especially when these low NIHSS scores correspond to so-called non-disabling deficits, such as sensory deficits, facial palsy, or dysarthria.

However, recent papers reported that patients with minor strokes do not always follow the expected favorable trajectory and are still disabled (14, 15). Approximately one-third of the patients have been described to show poor outcome at 3 months, defined as mRS ≥ 2 [see Table 1 of Khatri et al. (16)]. Indeed, the NIHSS score not only fails to capture some deficits that could affect the functional outcome in patients, such as executive functioning and other cognitive abnormalities but also hand dexterity or gait disturbances (8, 17).

In this study, we found a higher proportion of patients who did not return to their pre-stroke level after 3 months. This high percentage of poor outcomes highlights the importance of ongoing Randomized Controlled Trials that aim to investigate the benefit of more intense treatment of patients with minor strokes, especially when combined with large vessel occlusion (18, 19).

### MoCA: A Pivotal Assessment

The entry point of our decision tree is the MoCA with a cutoff of 26 points. It is now well established that patients scoring below 26 points are considered cognitively impaired (9). This highlights the importance of evaluating cognitive deficits and their impact on the level of independence in minor strokes. In Jaillard et al. 91.5% of the patients with stroke had deficits in at least one cognitive domain, and 73.4% of them had severe deficits when examined within the first month post-stroke (20). They had episodic or working memory, executive, and instrumental function disturbances, which largely explained why they were not able to perform their ADLs.

In our study, when MoCA scored below 26 points, the next step was to consider age to determine the outcome. Patients younger than 65 years were predicted to have a poor outcome. If they were 65 years or older, their outcome depended on the score of the MoCA with a cutoff of 24 points.

It is not surprising that patients with a MoCA score <26 and age <65 years did not return to their pre-stroke level of activities. Indeed, in patients <65 years, returning to their usual activities meant they continue to be full-time employed (as 65 years is the age cutoff for retirement in France as well as in Switzerland). We think that employed patients with a MoCA <26 were not able to return to work full-time because of the post-stroke cognitive decline. This is in line with the study of Van Der Kemp et al. (21), where half of the patients did not return to the pre-stroke occupational situation. With regard to factors predicting returning to work in their study, global cognitive functioning assessed by MoCA was associated with returning to work within 1 year as well as depression, which we did not investigate separately in our study (21). Indeed, fatigue and psychological impairment, such as anxiety, posttraumatic stress disorder, and depression, may negatively impact the outcomes (22). This corresponds to the so-called ≪hidden disability≫ or ≪invisible disability≫ described in the literature (23, 24).

In our study, patients older than 65 years were mostly retired (42/46, 91% for the construction cohort). Their usual activities were likely less demanding. However, when MoCA is less than 24 points this could be stroke-induced or a feature of pre-stroke cognitive impairment, probably not detected before.

When MoCA was normal (≥26), the NIHSS score at discharge pre-dicted the 3-month outcome in our analysis. When discharge NIHSS score was 0, patients reached their pre-stroke level. This confirms that when there were no other symptoms, cognitive functioning was crucial for returning to the pre-stroke activities. However, when the NIHSS score was >0, meaning that there was still a symptom measurable on this score, the outcome depended on the occupational status of the patient. Patients who were working were less likely susceptible to return to work.

### Errors of Prediction

Errors of prediction represented 20% of the construction cohort (*n* = 21) and 28% of the validation cohort (*n* = 28). Most errors occurred in the following node: ≪MOCA <24≫ and ≪age ≥ 65 yo≫. One possible explanation would be that MoCA scores may be lower as a result of normal aging without consequences on daily life activities (25). Given the small sample size, we could not perform a robust analysis to compare the patients correctly classified vs. wrongly classified in this node, but we noticed that the patients wrongly classified (predicted with a poor outcome unless they returned to their usual pre-stroke activities) had lesser motor symptoms (9 vs. 24%, *p*: 0.03) and were more independent before the stroke event. Indeed, the subgroup analysis presented in the Supplementary Material, restricted to patients with a pre-stroke mRS 0–1, demonstrated a similar accuracy between the construction and the validation cohorts (80 and 82% respectively) with a similar error rate.

With regards to the distribution of the error rate (false positive vs. false negative patients), from a patient's perspective, it is better when the outcome is predicted worse than it actually turned out to be, since the allocation of therapy could be more intense than finally needed, better than underestimated and not provided as needed. In terms of health care expenses, however, resources might be used more than needed and thus more expensive than needed if false-negative rate is higher than false-positive rate (26).

### Limitations

This study has several limitations. First, several prognostic factors were measured in a dichotomous way, which may have resulted in some loss of information. It is possible that a more comprehensive way of assessing these factors would have led to other results in our analysis. Second, we did not screen depression or fatigue in our patients. Post-stroke depression, fatigue, and cognitive dysfunction are closely associated with outcome, which may confound the present results. However, when depression was suspected at the acute phase, our French and Bernese patients received selective serotonin reuptake inhibitors (SSRIs) for three months until the next follow-up. Finally, aphasia was not entered as an independent predictor in our CART analysis. Nevertheless, aphasia when the present was not severe as (i) these patients were home discharged and (ii) we could perform the MoCA score in all patients.

In conclusion, this decision tree may help clinicians tailor planning of discharge in minor strokes. Indeed, in patients predicted ≪not returning to their usual prestroke activities≫, a better evaluation during the length of stay and a stronger follow-up (reinforcement of supportive services, outpatient rehabilitation) could lead to better outcomes.

## Data Availability Statement

The raw data supporting the conclusions of this article will be made available by the authors, without undue reservation.

## Ethics Statement

The studies involving human participants were reviewed and approved by CER-SU Ethics Committee, France and the Canton of Bern Ethics Committee, Switzerland. Written informed consent for participation was provided in Bern and was not required for this study in France in accordance with the national legislation and the institutional requirements.

## Author Contributions

MHo and CR wrote the manuscript. All other authors were involved in drafting the manuscript and revising it critically for important intellectual content. CC, LG, AL, and FB were responsible for the experimental work. MHo is the statistician of the study. All authors contributed to the article and approved the submitted version.

## Funding

The research leading to these results has received funding from Investissements d'avenir ANR-10-IAIHU-06.

## Conflict of Interest

The authors declare that the research was conducted in the absence of any commercial or financial relationships that could be construed as a potential conflict of interest.

## Publisher's Note

All claims expressed in this article are solely those of the authors and do not necessarily represent those of their affiliated organizations, or those of the publisher, the editors and the reviewers. Any product that may be evaluated in this article, or claim that may be made by its manufacturer, is not guaranteed or endorsed by the publisher.
