# Supplementary material for: Cognitive Status Predicts Return to Functional Independence After Minor Stroke: A Decision Tree Analysis
Source: Front Neurol. 2022 Feb 17;13:833020. doi: 10.3389/fneur.2022.833020 (PMC8891604; doi:10.3389/fneur.2022.833020)
Supplement: Supplementary file 1 [file Data_Sheet_1.docx]

**SUPPLEMENTARY MATERIAL**

**A decision tree predicting whether patients are returning to their usual activities after a minor stroke**

**Methods**

**CART analysis**

CART is a predictive model which explains how an outcome variable can be predicted based on other values without *a priori* assumptions on which explanatory variables to include, or their order in the tree. The CART output is a decision tree where each fork is a split in a predictor variable and each end node contains a prediction for the outcome variable. To be easily used in clinical practice, the CART analysis should have a maximum tree depth of 3 and minimum terminal node size of 3 cases.

Here, the target outcome corresponds to patients with a mRS 0 or 1 (as a binary variable) 3-months after a stroke. The explanatory variables entered in the CART analysis were age, gender, pre-stroke mRS, affected hemisphere, type of stroke, presence of motor or sensory symptoms, occupational status, household situation, need of assistance (technical, human or neither), MoCA at discharge, NIHSS score at admission and at discharge, and mRS at discharge. Before entering these variables into the CART analysis, we performed a Principal Component Analysis to identify if some of these variables covaried together and could be removed. The correlation circle (Supplementary figure 1) indicated that NIHSS at admission and discharge covaried together as well as pre-stroke mRS and need of assistance. Thus, admission NIHSS and need of assistance were removed from the CART analysis, because of their redondance and the decision tree was generated.

**Supplementary Figure 1: Correlation circle from the PCA analysis**

| 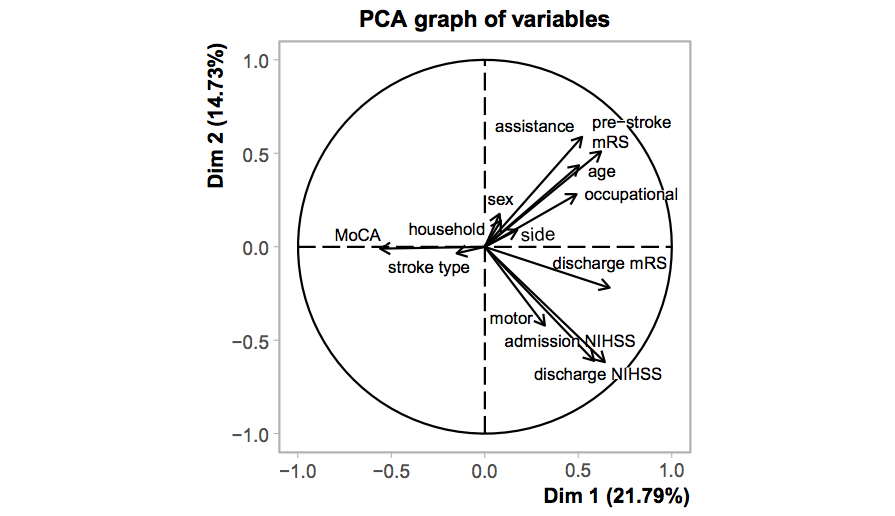 |
| --- |

**Results**

Analysis on patients with a pre-stroke mRS of 0-1

As the number of patients with a pre-stroke mRS >1 was higher in the Bernese (n=48) vs. the French cohort (n=8), we did a subgroup analysis in both cohorts for patients with a pre-stroke mRS 0-1. Baseline characteristics were reported in Supplementary Table I

At 3-months, 60 patients (61.8%) and 30 patients (57.7%) returned to their routine life (mRS 0-1) in the construction and validation cohorts respectively. Baseline characteristics for the two cohorts are summarized in Supplementary Table 1.

Supplementary Table 1. **Characteristics of the cohorts**

|  | **Construction cohort**  **N=97** | **Validation cohort**  **N=52** |
| --- | --- | --- |
| Age (years) | 64.5±15.7 | 66.3±14.8 |
| Gender, Males (n,%) | 58 (59.7%) | 33 (63.4%) |
| Cardiovascular risk factors  Hypertension  Hypercholesterolemia  Diabetes  Tobacco | 24 (24.7%)  20 (20.6%)  16 (16.5%)  7 (7.2%) | 28 (53.8%)  27 (51.9%)  14 (26.9%)  15 (28.8%) |
| Household situation  not living alone/living alone but proxies/ | 90/7/0  92.7% / 7.3%/ 0% | 32/19/1  61.5%/36.5%/2% |
| Occupational status  Employed/retired/unemployed | 43/48/6  44% / 49% / 5% | 19/31/2  37% / 60% / 3% |
| Admission NIHSS score | 1.3±1.3 | 1.6±1.7 |
| MoCA score | 23.3±4.7 | 25.1±3.6 |
| Discharge NIHSS score  - mean/SD  - N, % patients with a score of 0 | 0.8±1.1  53 (54.6%) | 1.4±1.9  22 (42.3%) |

In the construction cohort, the prediction accuracy for a favourable outcome was 80% (78/97), sensitivity was 71.4% and specificity was 91%. Errors of outcome prediction represented 20% of the patients. Three (3%) were false positive (predicted as a favourable outcome but did not reach it) and 16 (16%) were false negative (predicted as poor outcome but recovered better than expected).

In the validation cohort, the decision tree applied to this population reached a 82.7% (43/52) accuracy, 86.7% sensitivity (26/30) and 77.3% specificity (17/22). False positive and negative rates were 7.7% and 9.6% respectively.

It is important to note that the decision tree, even if pre-stroke modified Rankin scale was not retained during the construction of the three, is sensitive to it. Accuracy in the validation cohort is higher after the removal of patients dependent before the stroke event. Moreover, the rates of false positive and false negative cases were similar between the two cohorts.
